# Photoinduced spontaneous free-carrier generation in semiconducting single-walled carbon nanotubes

**DOI:** 10.1038/ncomms9809

**Published:** 2015-11-04

**Authors:** Jaehong Park, Obadiah G. Reid, Jeffrey L. Blackburn, Garry Rumbles

**Affiliations:** 1 National Renewable Energy Laboratory, Chemistry and Nanoscience Center, 15013 Denver West Parkway, Golden, Colorado 80401, USA; 2 Renewable and Sustainable Energy Institute, University of Colorado at Boulder, Boulder, Colorado 80309, USA; 3 Department of Chemistry and Biochemistry, University of Colorado at Boulder, Boulder, Colorado 80309, USA

## Abstract

Strong quantum confinement and low dielectric screening impart single-walled carbon nanotubes with exciton-binding energies substantially exceeding *k*_B_*T* at room temperature. Despite these large binding energies, reported photoluminescence quantum yields are typically low and some studies suggest that photoexcitation of carbon nanotube excitonic transitions can produce free charge carriers. Here we report the direct measurement of long-lived free-carrier generation in chirality-pure, single-walled carbon nanotubes in a low dielectric solvent. Time-resolved microwave conductivity enables contactless and quantitative measurement of the real and imaginary photoconductance of individually suspended nanotubes. The conditions of the microwave conductivity measurement allow us to avoid the complications of most previous measurements of nanotube free-carrier generation, including tube–tube/tube–electrode contact, dielectric screening by nearby excitons and many-body interactions. Even at low photon fluence (approximately 0.05 excitons per μm length of tubes), we directly observe free carriers on excitation of the first and second carbon nanotube exciton transitions.

## Abstract (web summary)

Photoinduced carrier-generation in individual semiconducting single-walled carbon nanotubes is controversial. Here, the authors demonstrate that free carriers can be generated even in the absence of dissociating interfaces by performing time-resolved microwave conductivity on solutions of dispersed nanotubes.

## Graphical abstract

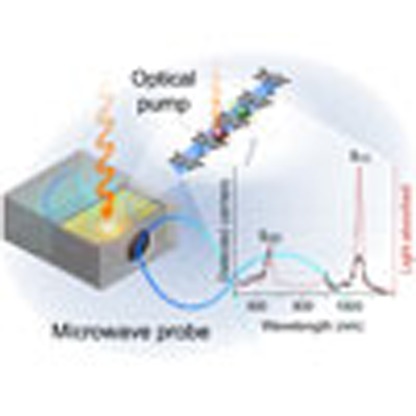

Photoinduced free-carrier generation in semiconducting single-walled carbon nanotubes (SWCNTs) has been controversial because of the substantial binding energy (hundreds of meV) of photogenerated excitons, coulombically bound electron–hole pairs^1^,^2^. Although a low probability of exciton dissociation is expected in SWCNTs, photoinduced carrier generation has been observed in neat SWCNT samples in a number of studies^3^,^4^,^5^,^6^,^7^,^8^,^9^,^10^,^11^,^12^. Most of these examples of carrier generation have been observed in solid-state samples featuring either tube–tube contacts or tube–electrode contacts. As these interfaces can likely serve as carrier-generation sites, they obscure the intrinsic properties of the individual nanotubes. Examples of these potential solid-state artefacts include heterogeneous chiralities of SWCNTs that may form type-I or type-II energy-level alignments in SWCNT bundles, electrostatic screening effects in SWCNT aggregates that can enhance free-carrier generation, SWCNTs on substrates in air that often become *p*-doped or potential morphological defects or contacts with electrodes in which electric fields can dissociate excitons^3^,^6^,^7^,^11^,^13^,^14^. Therefore, the prevalence of intrinsic carrier generation processes in well-isolated SWCNTs remains unclear. Importantly, such unintentional carrier generation can be detrimental to applications requiring long exciton lifetimes and high luminescence quantum yields, such as biological imaging and photovoltaics^15^,^16^,^17^.

Recent solution-phase photoluminescence and transient absorption studies have suggested that charges are generated at relatively high incident photon fluences in aqueous dispersions^9^,^18^. However, it is important to note that the exciton binding energy is predicted to decrease dramatically with increasing dielectric constant of the solvent (*ɛ*_r_(H_2_O)=80.1, ref. 19), and high incident photon fluences can reduce the exciton binding energy via screening, facilitate exciton–exciton collisions that lead to Auger-like photoionization or even damage the CNT surface to generate defects.

In this study we demonstrate that free charge generation takes place in individual SWCNTs suspended in toluene (*ɛ*_r_=2.38, ref. 19), even at ultra-low excitation fluences, which rules out both high dielectric and multi-exciton effects. We use flash-photolysis time-resolved microwave conductivity (*fp*-TRMC) on solution-phase samples, to study the complex photoconductance of individually suspended (7,5)-chirality-enriched SWCNTs ((7,5)-SWCNTs). The high sensitivity of this technique allows us to use excitation fluences that are much lower than has previously been possible (lower than 10^11^ photons per cm^2^); the lowest excitation fluences correspond to absorbed photon densities of approximately 0.04–0.06 photons per μm length of (7,5)-SWCNTs. We find that the low-fluence yield mobility product *Φ∑μ*, which is the product of charge-carrier generation efficiency *Φ* and the sum *∑μ* of electron and hole mobilities *μ*_e_+*μ*_h_ in isolated (7,5)-SWCNTs is approximately 0.17 and 0.4 cm^2^ V^−1^ s^−1^ following S_11_ and S_22_ photoexcitation, respectively.

## Results

### Dispersion of (7,5)-SWCNTs in toluene

**Figure 1:**
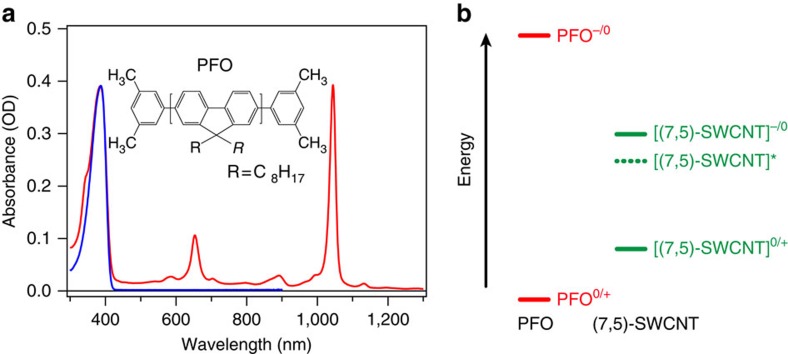
(7,5)-SWCNT dispersion wrapped with PFO polymer. (**a**) Electronic absorption spectra of (7,5)-SWCNTs suspended in toluene via polymer wrapping in PFO (in red) and of PFO polymer dissolved in toluene (in blue), respectively (see Methods for sample preparation). (**b**) Energy-level diagram illustrating that the PFO polymer and (7,5)-SWCNTs form a type-I heterostructure. A dotted line depicts the S_11_ state of [(7,5)-SWCNT]*.

The electronic absorption spectrum of (7,5)-SWCNTs suspended in toluene by poly[9,9-dioctylfluorenyl-2,7-diyl] (PFO) is shown in Figure 1a. Employing established methods with PFO as a dispersing agent^20^,^21^, we obtain highly (7,5)-chirality enriched SWCNT solution and remove excess PFO until a polymer:SWCNT mass ratio of approximately 4 is obtained (see Methods for full details of sample preparation). The distinctive S_11_ and S_22_ excitonic transitions of (7,5)-SWCNTs, peaking at 1,044 and 653 nm, respectively, confirm the purity of the (7,5)-SWCNT solution sample. In addition, electronic absorption data preclude the possibility that PFO is excited in the range from 450 to 1,200 nm. The energy-level diagram shown in Figure 1b illustrates that the PFO polymers^22^,^23^ and the (7,5)-SWCNTs^24^ form a type-I heterostructure and, therefore, it is evident that the (7,5)-SWCNT* state generated via S_11_ excitation should not exhibit electron/energy transfer from (7,5)-SWCNTs to PFO polymers, which is consistent with previous literature results^25^.

### Probing photoinduced free-carrier generation of SWCNTs

**Figure 2:**
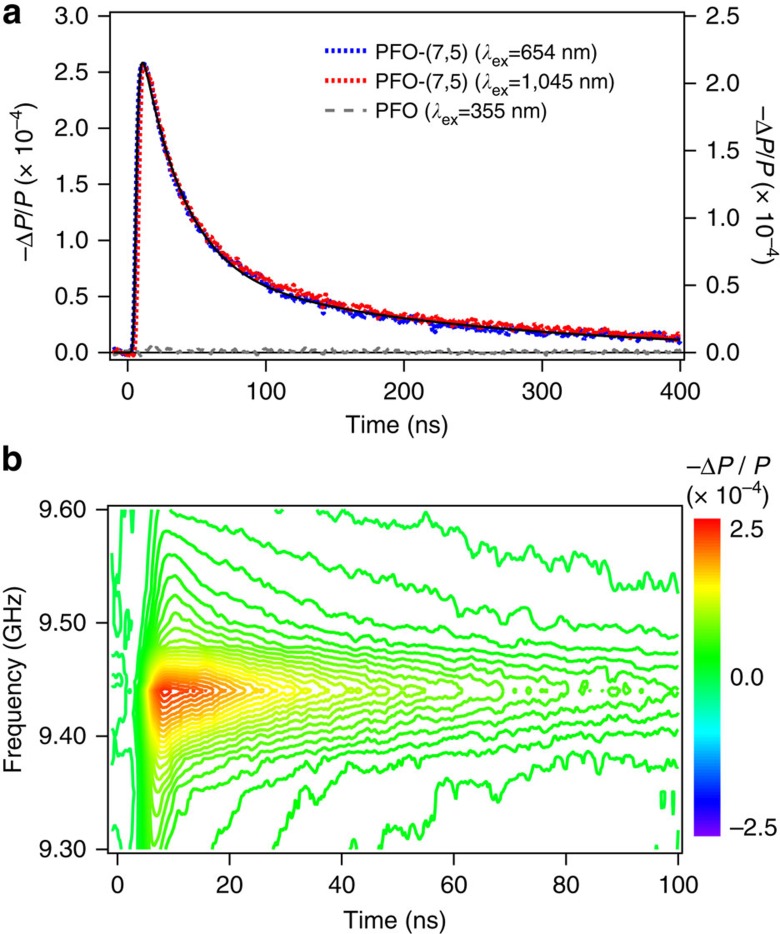
Phtoinduced free-carrier generation probed by microwave conductivity. (**a**) Representative time-resolved reflected microwave transients for (red and blue) (7,5)-SWCNTs suspended in toluene and (grey, left *y* axis) PFO dissolved in toluene. The SWCNT transients result from photoexcitation at either the S_22_ transition (blue, left *y* axis) or S_11_ transition (red, right *y* axis). (**b**) Frequency-dependent reflected microwave power transients for (7,5)-SWCNTs suspended in toluene, following excitation at S_22_. In **a**, a biexponential fit is displayed as the solid black line. Experimental conditions: the excitation photon fluence was approximately 1.0–1.5 × 10^12^ photons per cm^2^ for the data presented in **a** and 4.4 × 10^12^ photons per cm^2^ for **b**; 5 ns pulse width; room temperature.

We employ a cavity-based *fp*-TRMC technique to explore photoinduced free-carrier generation and recombination dynamics in the solution-phase (7,5)-SWCNTs. The use of the resonance cavity in *fp*-TRMC experiments allows us to operate at very low excitation photon fluences (approximately 10^10^–10^12^ photons per cm^2^) and to explicitly measure the complex conductivity, avoiding complications from many-body interactions such as exciton–exciton annihilation processes. In this regard, *fp*-TRMC has shown its versatility in determining the charge-carrier mobility, charge-carrier generation efficiency and subsequent carrier recombination dynamics for various conjugated polymer aggregates in solution, and thin-film layers, including organic layers or hybrid organic–inorganic layers^7^,^26^,^27^,^28^,^29^,^30^,^31^,^32^,^33^,^34^,^35^,^36^,^37^,^38^. Figure 2a displays representative time-resolved TRMC transients for (7,5)-SWCNTs suspended in toluene, as well as those for controls of PFO dissolved in toluene and neat toluene solvent in Supplementary Figure 1; the vertical axis is the change in microwave power reflected from the cavity at the resonance frequency. No reflected microwave transient signals are observed with either neat PFO dissolved in toluene (grey dashed line in Figure 2a, left *y* axis) or solvent alone (Supplementary Figure 1), showing that the change of microwave absorption is due solely to (7,5)-SWCNTs. *fp*-TRMC experiments for a variety of (7,5)-SWCNT concentrations (1.23–12.3 nM per μm length of (7,5)-SWCNTs) reveal that the transient decay dynamics are insensitive to concentration within this range (Supplementary Figure 2).

### Frequency-resolved microwave conductivity measurements

The details of the solution-phase *fp*-TRMC experimental setup and discussion are described in Supplementary Figures 3–6 and Supplementary Methods, and the theoretical background of *fp*-TRMC can be found elsewhere^28^,^29^,^39^,^40^. In brief, in the most general terms, *fp-*TRMC experiments measure the time evolution of the complex dielectric constant *ɛ* of the sample after photoexcitation. The present experiments are conducted with the sample mounted in a microwave cavity and the complex dielectric constant of the sample is calculated from the cavity resonance characteristics. Changes in the real part of the dielectric constant lead to a shift in the resonance frequency, whereas the imaginary part determines microwave power loss in the cavity. Charges photogenerated in the sample (photoconductivity) can contribute to both the real and the imaginary parts of the dielectric constant depending on their mobility and degree of confinement^40^. Conductivity can be expressed in terms of dielectric constant as:


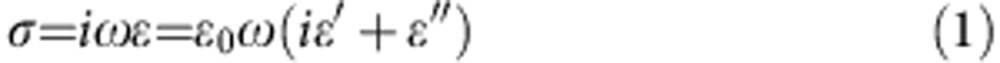


where *σ*, *ω*, *ɛ*_0_, *ɛ′* and *ɛ″* represent the complex conductivity, the radian frequency of the microwave electric field, the vacuum permittivity, and the real and imaginary parts of the dielectric constant at frequency *ω*, respectively. Thus, the real part of the conductivity is identified with the imaginary part of the dielectric constant—microwave absorption. An important caveat here is that real conductivity is only one of two possible components of the loss term. Dielectric loss can also contribute, as when molecular dipoles re-orient in the field. These two loss mechanisms are indistinguishable in the TRMC experiment and we rely on arguments later in the text, to show that a significant dielectric loss contribution to the signals we observe is unlikely.

*fp*-TRMC measurements made only at a single resonance frequency cannot distinguish between the real and imaginary parts of the conductivity, because a frequency shift simply modulates the reflected power, much as a change in power absorption would^41^. Frequency-dependent measurements are therefore necessary to identify the origin of the transient signals in (7,5)-SWCNTs. A set of reflected power transients were collected at 13 different microwave frequencies, spanning the cavity resonance curve, following S_22_ excitation of a (7,5)-SWCNT solution. Figure 2b and Supplementary Figure 7 show contour plots of frequency-dependent reflected microwave power transients generated from these data and exhibit a negligible frequency shift as a function of time, implying that the transient signals obtained on resonance are dominated by a change in the real conductivity of the sample. These results conclusively demonstrate that photoexcitation of (7,5)-SWCNTs generates some amount of mobile free carriers.

### Transient photoconductance decay

Under both S_11_ and S_22_ excitation conditions, microwave transient decay dynamics normalized at the peak are indistinguishable as shown in Figure 2a. In a previous study of SWCNT thin films, the photoconductance Δ*G* signal, which is proportional to the reflected microwave power −Δ*P*/*P*, decayed by approximately 90% within the first 10 ns (ref. 7). In our current solution-phase *fp*-TRMC measurements, the transient signals persist much longer. Both transient decay profiles are fitted using a biexponential function with time constants *τ*_i_ and associated amplitudes *a*_i_ of *τ*_1_=27 ns (*a*_1_=0.75) and *τ*_2_=212 ns (*a*_2_=0.25), and yield the average lifetime *τ*_avg_ of 161 ns, from *τ*_avg_=Σ_i_*f*_i_*τ*_i_, where *f*_i_ is the fractional contribution of each time constant, which is (*a*_i_*τ*_i_)/Σ_j_*a*_j_*τ*_j_. The different transient decay behaviour between solution-phase individualized SWCNTs and thin-film SWCNTs suggest that inter-tube junctions in SWCNT thin films possibly facilitate carrier recombination by serving as recombination sites^42^. In contrast, the longer-lived solution-phase TRMC transient decay dynamics probably represent more intrinsic intra-tube carrier-recombination dynamics, as inter-tube contact is prohibited in the highly individualized SWCNTs.

### Photoconductance action spectrum

**Figure 3:**
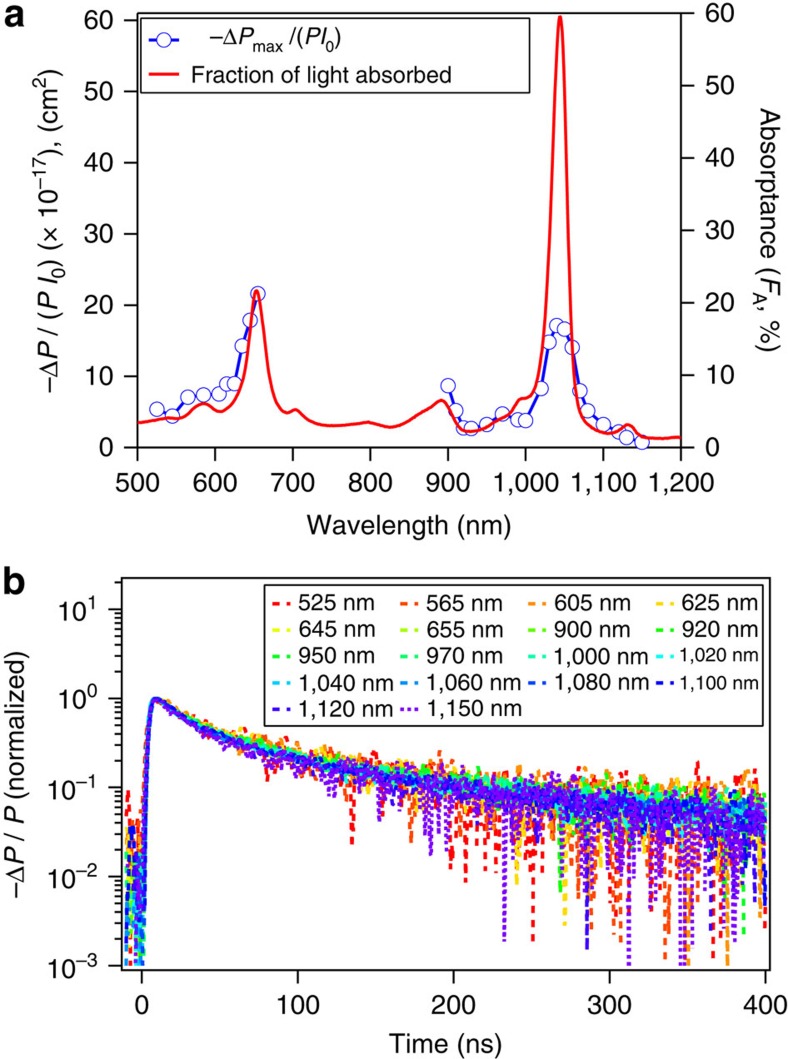
Photoconductance action spectrum and excitation wavelength dependence of photoconductance transients. (**a**) Action spectrum for peak reflected microwave signal (Δ*P*/*P*, end of pulse) normalized by the excitation photon fluence (*I*_0_) for (7,5)-SWCNTs suspended in toluene (blue, left *y* axis). Electronic absorptance spectrum of (7,5)-SWCNTs suspended in toluene is overlaid for comparison (red, right *y* axis). (**b**) Normalized reflected microwave transients decay for a variety of excitation wavelengths noted in the figure. Experimental conditions: *I*_0_<5 × 10^12^ photons per cm^2^; room temperature.

Figure 3a displays the action spectrum of the peak reflected microwave power for (7,5)-SWCNTs suspended in toluene, which examines the correlation of carrier generation with excitation wavelength. Photoconductance values in the action spectrum are taken at low photon fluence (*I*_0_<5 × 10^12^ photons per cm^2^) and are normalized for excitation photon fluence. The shape of the action spectrum closely matches the absorptance spectrum of (7,5)-SWCNTs, demonstrating further that the origin of the photoconductance is indeed (7,5)-SWCNTs. Equations (2) and (3) show the relation between the reflected microwave power and the carrier-generation yield.


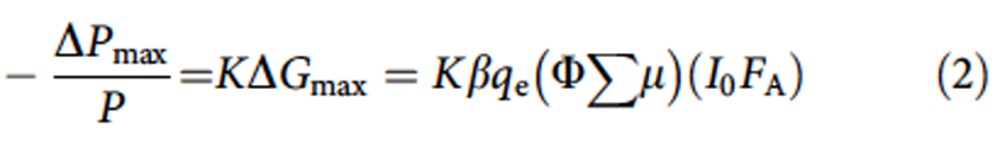



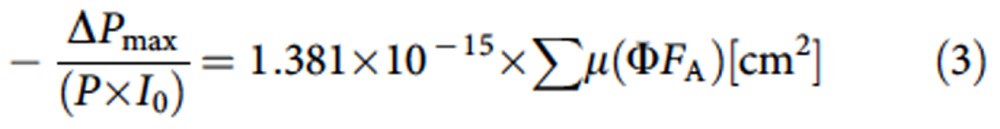


In equations (2) and (3), *I*_0_ (photons per cm^2^ per pulse) is the excitation photon fluence, *F*_A_ is the fraction of light absorbed at the excitation wavelength (absorptance), *K* (Ω) is a sensitivity factor, determined as 2,400 from the cavity resonance characteristics and the dielectric properties of the medium (see Supplementary Methods for evaluation of *K* factor), *β* is the ratio between the long and short axes of the sample cross-section that is perpendicular to the microwave propagation vector, *q*_e_ (C) is the elementary charge and *∑μ* is the sum of electron and hole mobilities. The reflected microwave power normalized by the incident photon fluence −Δ*P*/(*PI*_0_) is proportional to *ΦF*_A_, as the mobilities can be assumed constant. Therefore, by comparing −Δ*P*/(*PI*_0_) with the absorptance spectrum, we can extract the relative carrier-generation yield for S_11_ and S_22_ excitation. Interestingly, the action spectrum comparing *ΦF*_A_ near S_11_ and S_22_ transitions (Figure 3a) shows that the carrier-generation quantum yield *Φ* under S_22_ excitation is about three times higher than that under S_11_ excitation.

Recently, Kumamoto *et al.*^11^ reported photocurrent with S_22_ excitation for an individual (10,6)-SWCNT grown on a Si substrate. Although the excitation wavelengths of their experiments reside within the S_22_ spectral domain, they clearly identified the presence of photocurrent with S_22_ excitation and non-zero conductivity even with zero applied bias, suggesting that the S_22_ exciton dissociation is a spontaneous process. In addition, Kazaoui *et al.*^10^ observed qualitatively higher photocurrent quantum yield with S_22_ excitation over that with S_11_ excitation for a (7,5)-SWCNT thin film. Likewise, as we probe photoinduced carrier generation in a low dielectric solvent, our results also suggest that exciton dissociation in (7,5)-SWCNTs is unlikely due to an electric field and more likely a spontaneous process in SWCNTs.

To rule out the possibility that the PFO wrapping the nanotube provides a locally higher dielectric constant, we have performed microwave cavity resonance measurements as a function of PFO:toluene mass ratio and compared them with analogous experiments where a higher dielectric constant solvent is added to the toluene. If the guest molecule added to neat toluene possesses a different dielectric constant from toluene, then the resonance frequency of the loaded microwave cavity will shift in response. The fact that the cavity resonance position does not shift detectably as PFO is added, in contrast to many of the more polar guest solvents, suggests that the PFO polymer has essentially the same dielectric constant as the toluene and does not provide a high local dielectric environment around the nanotubes (see Supplementary Figure 8 and Supplementary Methods for microwave cavity resonance measurements as a function of a guest molecule:toluene mass ratio).

**Figure 4:**
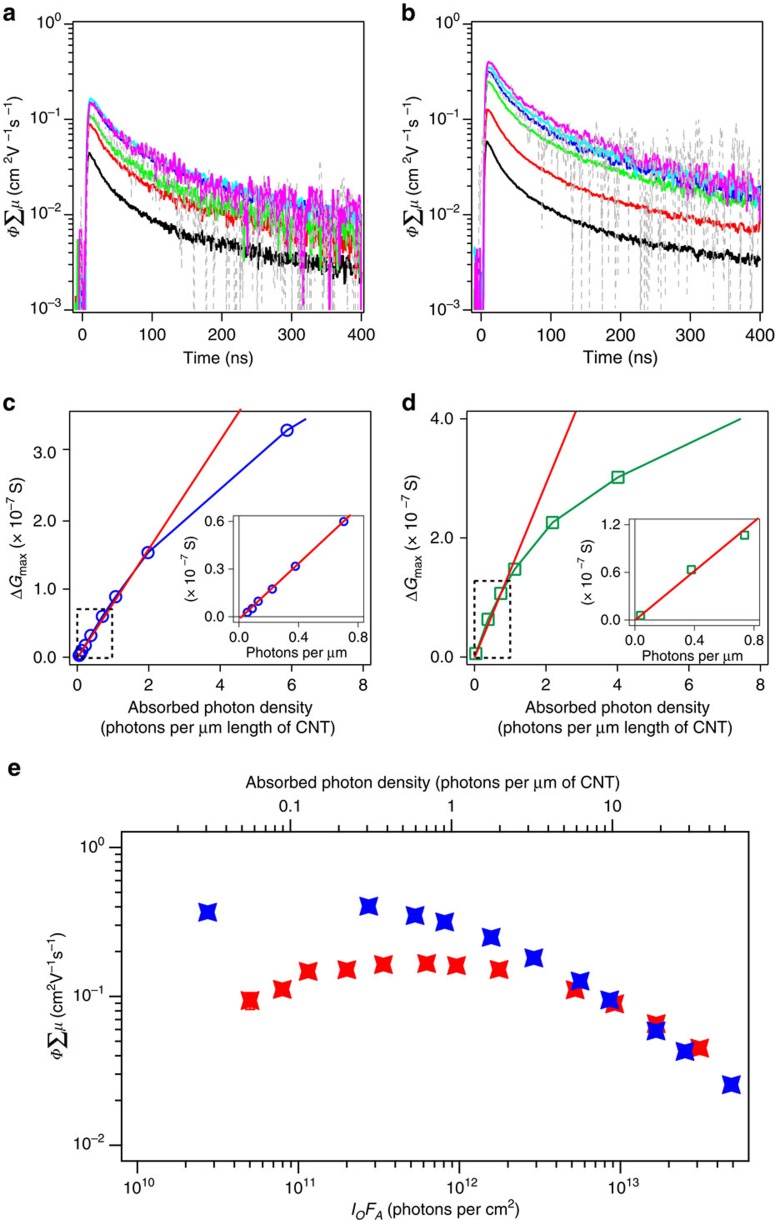
Excitation fluence-dependent photoconductance. (**a**,**b**) The dependence of the yield mobility product *Φ∑μ* transient decays on excitation photon fluence (*I*_0_) for excitation of the (**a**) S_11_ transition (black, red, green, blue, cyan, purple and grey: 3,470 × 10^10^, 1,030 × 10^10^, 584 × 10^10^, 197 × 10^10^, 70.0 × 10^10^, 22.3 × 10^10^ and 8.87 × 10^10^ photons per cm^2^, respectively) and (**b**) S_22_ transition (black, red, green, blue, cyan, purple and grey: 4,520 × 10^10^, 1,520 × 10^10^, 427 × 10^10^, 220 × 10^10^, 144 × 10^10^, 74.1 × 10^10^ and 7.41 × 10^10^ photons per cm^2^, respectively). (**c**,**d**) The peak of reflected microwave transients, Δ*G*_max_ (end of pulse), evincing the linearity of peak reflected transient signals below the absorbed photon density of approximately 0.8 photon per μm length of (7,5)-SWCNTs for both (**c**) S_11_ and (**d**) S_22_ transitions. The red solid lines represent a linear function. (**e**) *Φ∑μ* as a function of absorbed photon fluence for (7,5)-SWCNTs suspended in toluene with exciting at (blue) S_22_ or (red) S_11_ transitions, respectively. The top *x* axis corresponds to the absorbed photons per μm length of (7,5)-SWCNTs for given *I*_0_*F*_A_ (bottom *x* axis).

As the action spectrum of the peak reflected microwave power (normalized for excitation photon fluence) near the S_22_ transition of (7,5)-SWCNTs appears to match its excitonic features, no other intermediate state seems to be involved between photoexcitation and S_22_ exciton formation. This correlation suggests that carriers are produced from S_22_ excitons and carrier generation seems to compete with the S_22_→S_11_ internal conversion process that is known to be very fast (faster than 50 fs)^43^. Given the exciton binding energy (approximately 0.4 eV) for (7,5)-SWCNTs^1^,^2^, the continuum states for the S_11_ exciton lie below the lowest unoccupied molecular orbital of the S_22_ state. Autoionization of S_22_ excitons via populating a vibronically hot S_11_ state or free-carrier continuum states has been proposed for the charge-generation mechanism previously^5^,^44^, and it should be noted that in our action spectrum the carrier-generation yield with excitation at the S_11_ phonon side band (approximately 900 nm) appears to be comparable to that of S_22_ excitation. Figure 3b demonstrates identical microwave-transient decay profiles for widely varying excitation wavelengths from S_11_ to energies higher than S_22_. The similarity of these transients suggests that the initial photoproducts from either S_11_ or S_22_ excitation do not have an impact on the carrier decay dynamics, implying that the mobile carriers generated from photoexcitation are the same species, regardless of excitation energy. It should be noted that, although these <1-nm diameter SWCNTs should have S_11_ exciton binding energies of approximately 0.4 eV^1^,^2^, carrier generation following S_11_ excitation even at lower excitation fluences (lower than 10^11^ photons per cm^2^; Figure 4a) is unambiguously observed, although the yield is one-third of that observed for S_22_ excitation.

### Excitation fluence-dependent yield-mobility product

The figure of merit extracted from *fp*-TRMC using equations (2) is the product *Φ∑μ* of the free-carrier yield *Φ* and the sum of mobilities *∑μ*, which is proportional to the photoconductance Δ*G* normalized by the absorbed photon fluence *I*_0_*F*_A_. Figure 4a,b (and further in Supplementary Figure 9) displays the time-resolved *fp*-TRMC results of toluene-suspended (7,5)-SWCNTs following S_11_ and S_22_ excitations, respectively, where the *y* axis has been converted to *Φ∑μ*. On photoexcitation at the S_11_ transition, *Φ∑μ* values can be as high as approximately 0.17 cm^2^ V^−1^ s^−1^ in a variety of excitation photon fluences *I*_0_ ranging from 5.6 × 10^10^ to approximately 3.5 × 10^13^ photons per cm^2^, corresponding to an absorbed photon density of approximately 0.056–35 photons per μm length of (7,5)-SWCNTs (*Φ∑μ* transient data from 5.6 × 10^10^ photons per cm^2^ is provided in Supplementary Figure 9, see Supplementary Methods for the calculation of absorbed photon density in (7,5)-SWCNTs). Assuming that one absorbed photon creates one exciton, the absorbed photon density per μm length of (7,5)-SWCNTs corresponds to the initial exciton population per μm length of (7,5)-SWCNTs (hereafter abbreviated as [Ex]_μm_). On the other hand, *Φ∑μ* values following excitation of the S_22_ transition are as high as approximately 0.4 cm^2^ V^−1^ s^−1^ with excitation fluences below 7.4 × 10^11^ photons per cm^2^, which is about 2.4 times higher than the maximum *Φ∑μ* observed for S_11_ excitation. As the carrier mobility can be assumed to be constant between S_11_ and S_22_ excitation, the 2.4 times higher *Φ∑μ* on photoexcitation of the S_22_ transition suggests the carrier-generation yield with S_22_ excitation is approximately 2.4 times higher than that with S_11_ excitation. This result is consistent with the *fp*-TRMC action spectrum results in Figure 3a.

Even with approximately three orders of magnitude fluence increase, the microwave transient decay dynamics appear to be insensitive to the exciton density, as shown in the normalized transient decay profiles in Supplementary Figure 9. This result infers that in this exciton density range (0.056–35 [Ex]_μm_ of (7,5)-SWCNTs), carrier recombination effectively occurs through a first-order process and the interactions between a carrier and a neighbouring carrier created from another exciton are negligible for carrier recombination. The absence of fluence dependence within our excitation conditions (*I*_0_ between approximately 8 × 10^10^ and 4000 × 10^10^ photons per cm^2^) under our very low exciton density presumably infers a geminate carrier recombination process. Considering that our TRMC technique is detecting only free carriers, this carrier recombination is best described as a secondary geminate recombination process. Secondary geminate recombination requires dissociation and separation of the charges initially bound as an exciton, whereby subsequent recombination is diffusion mediated. The fairly long average transient decay lifetime (approximately 160 ns) suggests that one carrier might be trapped and only the other carrier is mobile.

Although we cannot attribute previously observed all long-lived (above the ns time domain) excited population in solution-phase studies to free carriers^45^,^46^, using those yields (approximately 3–10%) we can estimate the lower limit of free-carrier mobilities. At a conservative estimate for free-carrier mobilities with 10% yield, assuming only one carrier species is mobile, 9 GHz free-carrier mobility of (7,5)-SWCNTs is calculated to be higher than 4 cm^2^ V^−1^ s^−1^ with S_22_ excitation. This estimated free-carrier mobility is comparable to the previous literature values of semiconducting SWCNT thin-film carrier mobilities that range from 1.3 to 2 cm^2^ V^−1^ s^−1^ (refs 7, 47). On the other hand, when using higher available literature values of SWCNT carrier mobility^48^, we can instead speculate the lower limit of the carrier-generation yield, which is 0.04% using approximately 1,000 cm^2^ V^−1^ s^−1^.

Figure 4c,d show that the maximum photoconductance Δ*G*_max_ extracted from the photoconductance peak intensity increases linearly with exciton density below approximately 0.8 [Ex]_μm_ of (7,5)-SWCNTs. These results imply that carrier generation in this low fluence regime (lower than 0.8 [Ex]_μm_) is not a bimolecular process, although we cannot rule out the possibility that at a higher excitation intensity exciton–exciton interactions could mediate exciton dissociation^7^,^9^.

Figure 4e compares *Φ∑μ* as a function of *I*_0_*F*_A_ with either S_11_ (red) or S_22_ (blue) excitation. At low *I*_0_*F*_A_, where many-body interactions such as exciton–exciton annihilation or exciton–carrier annihilation are negligible, and assuming carrier mobility is constant, then *Φ∑μ* should be independent of *I*_0_*F*_A_. As a result, *Φ∑μ* exhibits a plateau at sufficiently low *I*_0_*F*_A_ range, which has been observed in many other neat thin films or donor–acceptor systems^29^,^30^,^34^,^49^. This plateau suggests that photoinduced carrier generation is a pseudo first-order reaction at low fluence. Likewise, *Φ∑μ* exhibits a plateau at exciton density below approximately 0.8–1 [Ex]_μm_ and begins to decrease with *I*_0_*F*_A_ increase, as a result of many-body interactions^7^. It is important to note that transient signals are present even at approximately 0.06 [Ex]_μm_ of (7,5)-SWCNTs with the S_11_ excitation (much less than one exciton per SWCNT on average), suggesting that photoinduced carrier generation at these low fluences does not result from exciton–exciton interactions. Given the previous study showing electric-field dependence of photocurrent generation^6^, we conjecture that local electric fields present at tube ends or defects could induce exciton dissociation for the case of S_11_ excitation.

## Discussion

In conclusion, we use a solution-phase *fp*-TRMC measurement and individualized highly (7,5)-chirality-enriched SWCNT samples dispersed in toluene by the PFO. We probe photoinduced mobile carrier generation in highly isolated SWCNTs in a low dielectric solvent (toluene, *ɛ*_r_=2.38) at very low exciton densities (lower than 0.06 excitons per μm). Even under these mild conditions, we unambiguously observe photoconductance in well-isolated (7,5)-SWCNTs with a yield-mobility product of approximately 0.17 and 0.4 cm^2^ V^−1^ s^−1^ following S_11_ and S_22_ photoexcitation, respectively. The carrier-generation quantum yield with S_22_ excitation appears approximately 2.4 times higher than that with S_11_ excitation, suggesting that the autoionization of S_22_ excitons enhances the carrier-generation efficiency. In contrast, the transient decay dynamics are independent of excitation wavelength, suggesting that the mobile carriers generated from either S_11_ or S_22_ excitation undergo identical decay pathways, that is, trapping, recombination, etc. This study demonstrates that frequency-resolved solution-phase *fp*-TRMC is beneficial to interrogate carrier dynamics of SWCNTs, as selective monitoring of free carriers is achievable with relatively fast ns-time resolution, and photoinduced processes can be studied at extremely low excitation fluence conditions (lower than 10^11^ photons per cm^2^). In addition, in solution-phase *fp*-TRMC, morphology-mediated information can be avoided, to investigate intrinsic material properties and dynamics. With these advantages in mind, this frequency-resolved solution-phase *fp*-TRMC technique can be applied to a variety of other nanomaterial systems.

## Methods

### PFO-(7,5) SWCNTs purification and sample preparation

SWCNT powder was added to approximately 2 mg ml^−1^ PFO solution in toluene such that the final weight ratio between SWCNT powder and PFO in toluene becomes 1:2 and the solution was dispersed through tip sonication (1/2 in probe) for 30 min at 40% intensity (Cole-Palmer CPX 750) in a bath of cool (18 °C) flowing water. The dispersion was then centrifuged using an SW32Ti rotor (Beckman) at 13,200 r.p.m. and 20 °C for 10 min. The supernatant, containing highly (7,5)-enriched SWCNTs, was then collected. The (7,5)-SWCNT dispersion was then centrifuged at 24,100 r.p.m. and 0 °C for 20 h, to remove excess solution-phase (unbound) PFO and to concentrate the (7,5)-SWCNTs for the *fp*-TRMC experiments. In this case, the resulting supernatant (containing free solution-phase PFO polymer) was discarded and the pellet (containing the (7,5)-SWCNT material) was redispersed in toluene. After this process, the PFO:SWCNT mass ratio is approximately 4:1. To calculate PFO:SWCNT mass ratio, the PFO mass extinction coefficient was experimentally determined in toluene as 95.7 l g^−1^ cm^−1^ (ɛ_387nm_=37,000 M^−1^ cm^−1^ for fluorene repeating unit in toluene).

### *fp*-TRMC experiments

The details of *fp*-TRMC experimental setup and its theoretical background have been reported elsewhere^28^,^29^,^39^,^40^ and the full accounts of solution-phase *fp*-TRMC are provided in Supplementary Methods. A schematic instrumental layout is described in Supplementary Figure 3. Although the details of *fp*-TRMC apparatus have been reported previously^50^,^51^, several modifications to load a solution cell are necessary to carry out solution-phase *fp*-TRMC. The SWCNT solution sample is loaded in a custom-designed cuvette (5-mm beam path length), which is shown in Supplementary Figure 4. This cuvette is mounted in a pocket made of PTFE (poly tetra fluoro ethylene; Teflon) and the pocket is positioned at the brass base as shown in Supplementary Figure 4. For illuminating the cuvette, 13 holes are made in the waveguide with a diameter of 3.175 mm, to prevent leakage of microwaves. The details of the microwave resonance cavity characterization such as K-factor and resonance curve measurements are described in Supplementary Methods. The sample is optically excited through the pattern of 13 holes by an approximately 5-ns full width at half maximum laser pulse from an optical parametric oscillator (Continuum Panther), pumped by the 355 nm harmonic of an Nd:YAG laser (Continuum Powerlite) and sample photoconductance is measured by monitoring the transient change in microwave power absorption by the sample after the laser pulse. The excitation power is adjusted with a series of neutral density filters. To measure an incident excitation power through the 13-hole pattern, we manufacture a mask with a same pattern and mount on the laser power meter sensor.

## Additional information

**How to cite this article:** Park, J. *et al.* Photoinduced spontaneous free-carrier generation in semiconducting single-walled carbon nanotubes. *Nat. Commun.* 6:8809 doi: 10.1038/ncomms9809 (2015).

## Supplementary Material

Supplementary Information Supplementary Figures 1-9, Supplementary Methods and Supplementary References.
