# Supplementary material for: Photoinduced spontaneous free-carrier generation in semiconducting single-walled carbon nanotubes
Source: Nat Commun. 2015 Nov 4;6:8809. doi: 10.1038/ncomms9809 (PMC4667683; doi:10.1038/ncomms9809)
Supplement: Supplementary Information — Supplementary Figures 1-9, Supplementary Methods and Supplementary References. [file ncomms9809-s1.pdf]

Supplementary Figures

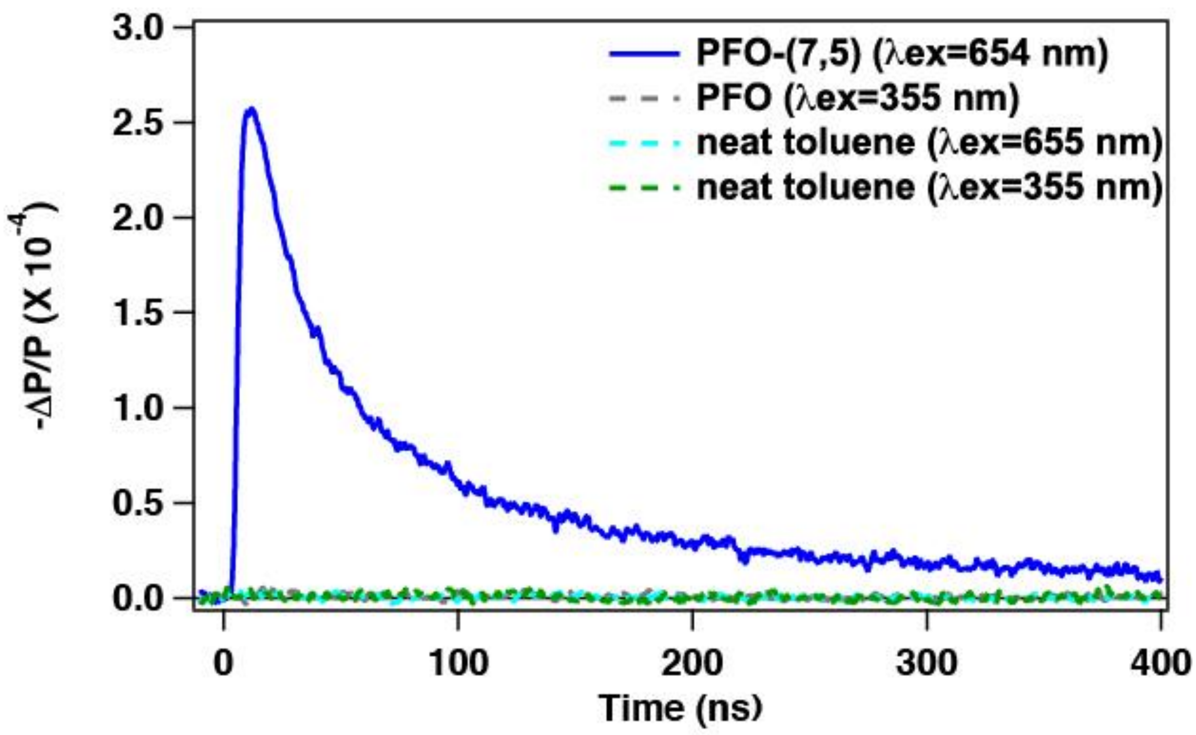

Supplementary Figure 1. Representative reflected microwave transients for (blue) (7,5)-SWCNTs suspended in toluene by wrapping with PFO, (gray) PFO dissolved in toluene, and (cyan and green) neat-toluene solvent with excitation wavelength noted. Experimental conditions: excitation photon fluence= ~1.5—3.0×10^{12} photons cm^{-2}; 5 ns pulse width; room temperature.

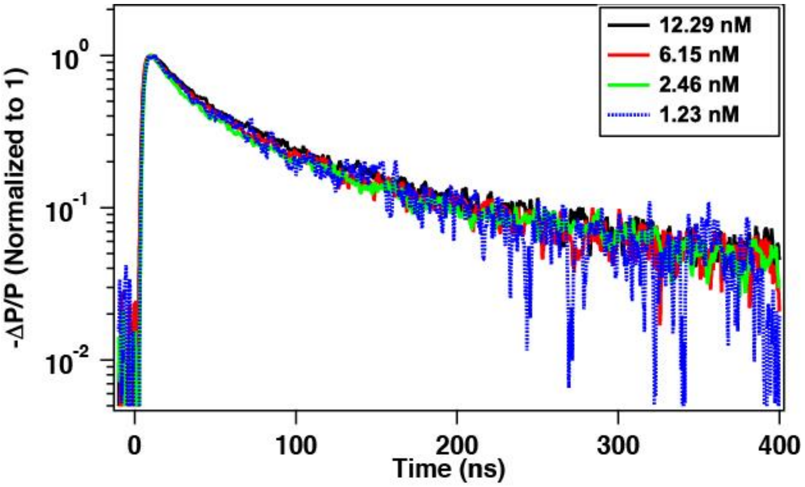

Supplementary Figure 2. Normalized reflected microwave transients for (7,5)-SWCNTs suspended in toluene by wrapping with PFO in a variety of (7,5)-SWCNT concentration noted. Experimental conditions: λ_{ex}= 645 nm, excitation photon fluence = ~1.1×10^{12} photons cm^{-2}; 5 ns pulse width; room temperature.

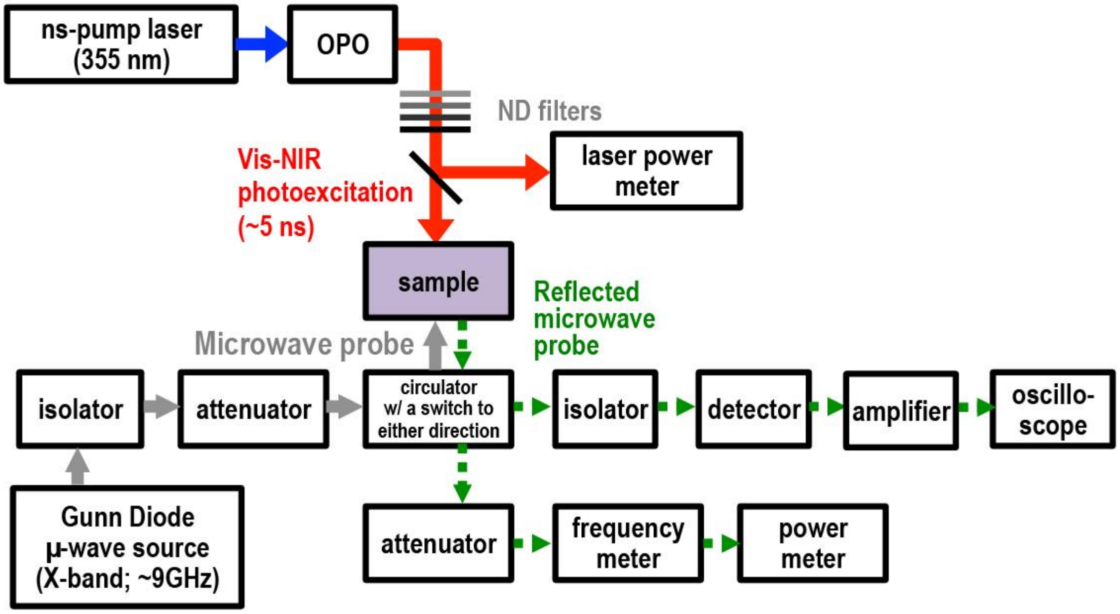

Supplementary Figure 3. Schematic representation of the flash photolysis time-resolved microwave photoconductivity (*fp*-TRMC) setup.

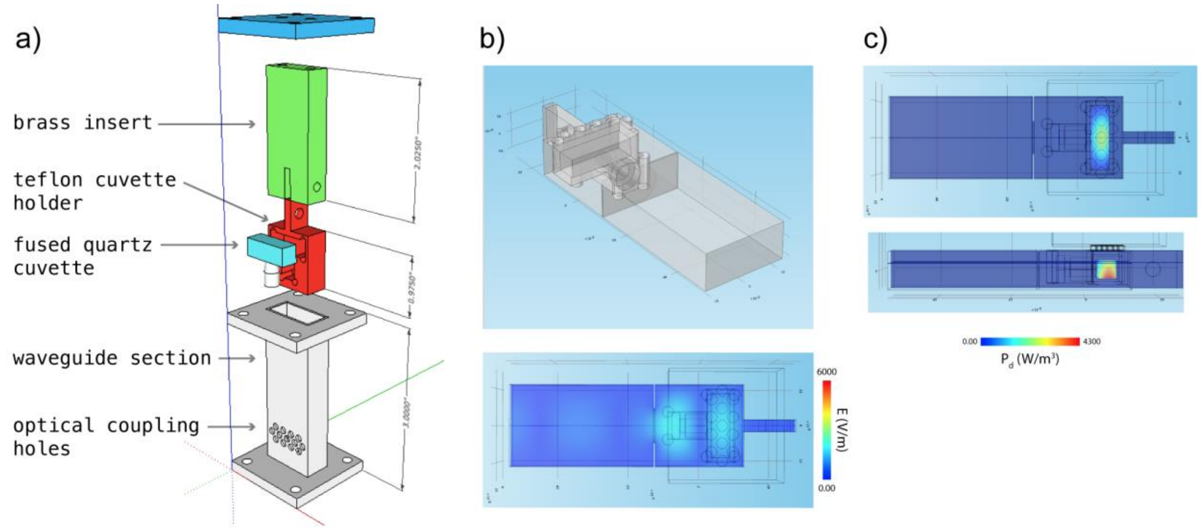

Supplementary Figure 4. a) An exploded schematic of the solution cavity configuration. b) (top) the solution cavity geometry as defined in COMSOL Multiphysics; (bottom) top view of the electric field distribution in a slice through the center of the cavity. c) Top and side views of the power dissipation density in the cavity, in this case dominated by dielectric loss in the toluene sample.

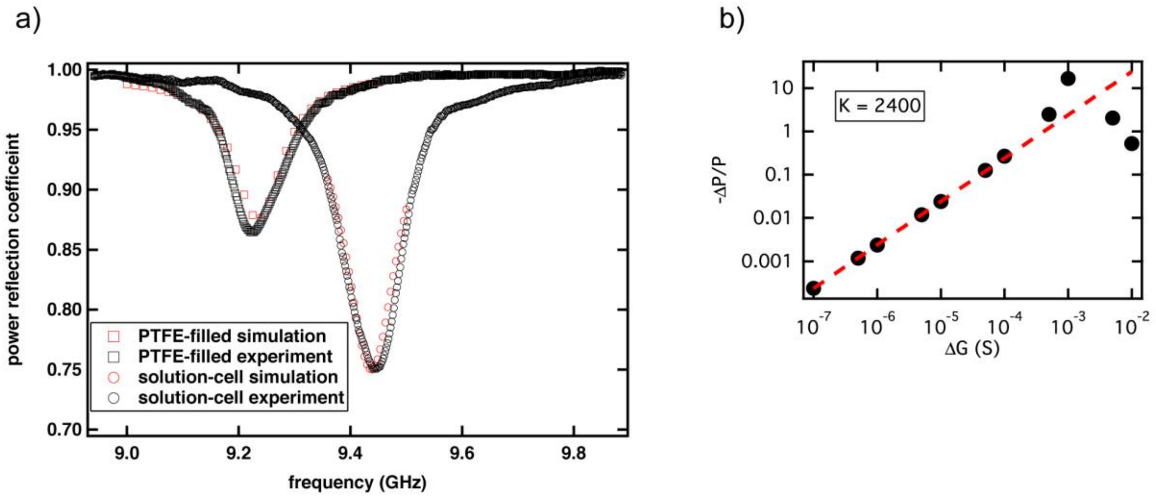

Supplementary Figure 5. a) Simulated and experimental resonance curves for a cavity filled with Teflon (PTFE, squares), and the full solution-cell configuration, where the cell was filled with toluene (circles). b) The ratio of change in reflected power to the initial reflected power vs. the change in sample conductance. The slope in the linear regime ($\Delta G \leq 1\times10^{-4}$) gives the sensitivity factor, K.

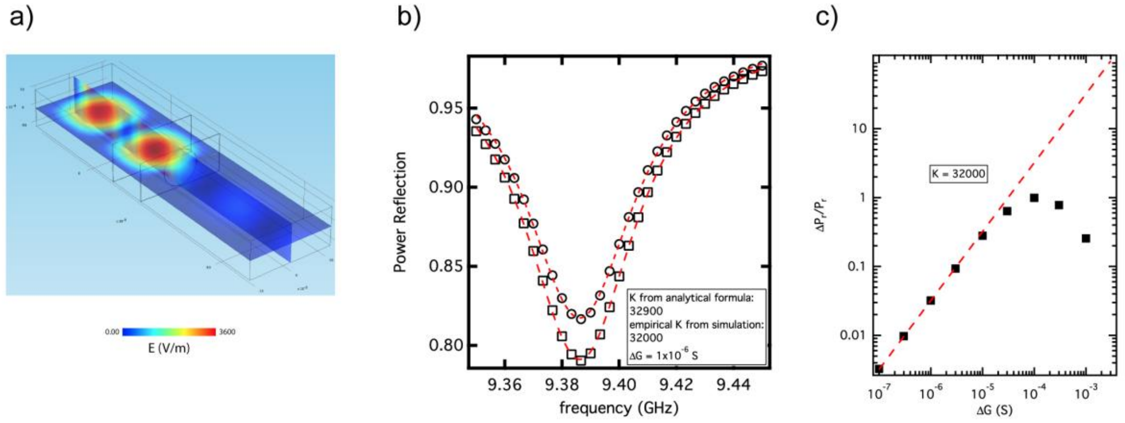

Supplementary Figure 6. a) The electric-field distribution for a simple rectangular TE_{101} resonant cavity, pumped on resonances at ~9.38 GHz. b) The resonance curves for the cavity pictured in a) plotted as the power reflection coefficient vs. frequency. The circles indicate the resonance curve for a sample with zero conductance, while the squares depict that for a sample with $\Delta G = 10^{-6}$ S. The red dashed lines are fits to a Lorentzian function, which yields Q = 200. c) The ratio of change in reflected power to the initial reflected power vs. the change in sample conductance. The slope in the linear regime ($\Delta G \leq 3\times10^{-6}$ S) gives the sensitivity factor, K.

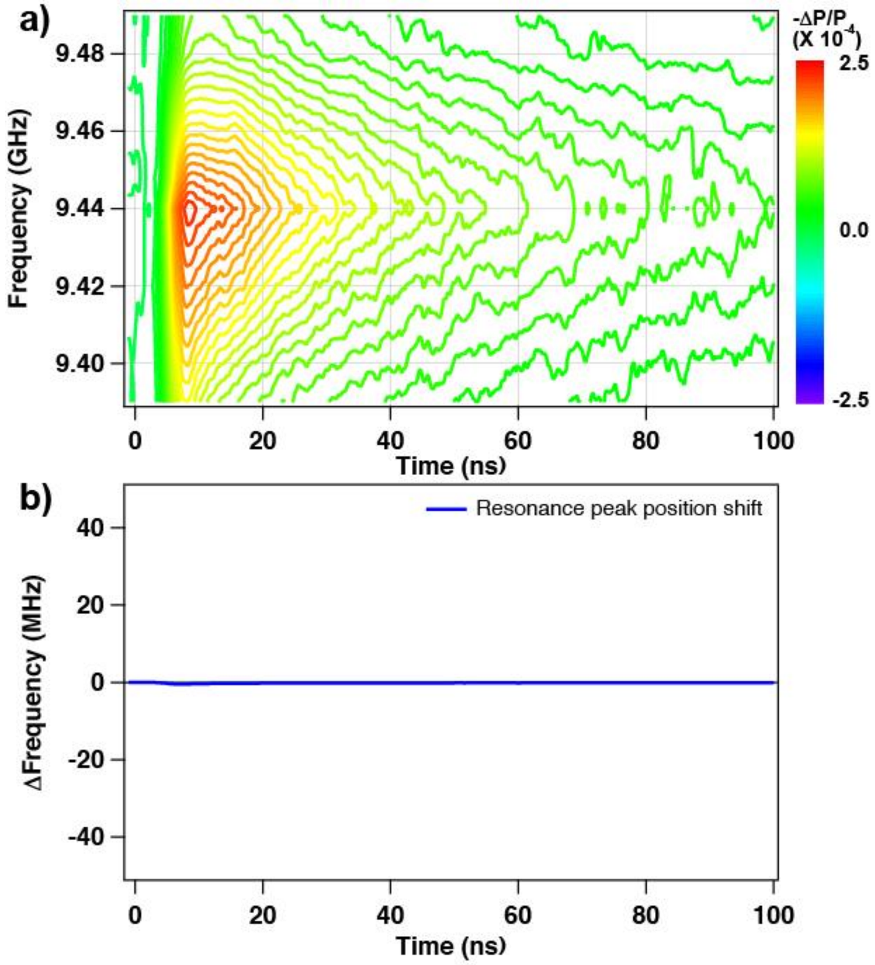

Supplementary Figure 7. a) Frequency dependent reflected microwave power transients for (7,5)-SWCNTs suspended in toluene, zooming in to the frequency window between 9.39 and 9.49 GHz, with exciting at S_{22}. b) Microwave cavity resonance frequency peak position shift as a function of time following photoexciting the S_{22} state for (7,5)-SWCNTs. Experimental conditions: λ_{ex}= 655 nm; excitation photon fluence= 4.4×10^{12} photons cm^{-2}; 5 ns pulse width; room temperature.

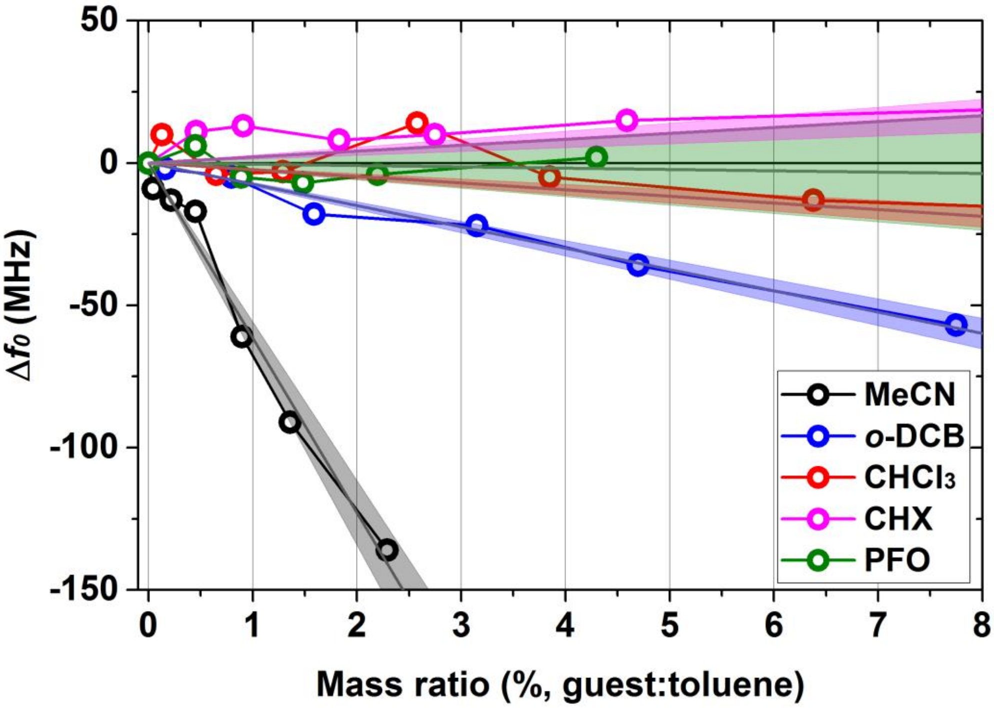

Supplementary Figure 8. The resonance frequency (f_0) measurements as a function of guest:toluene solvent mass ratio in the solution-phase *fp*-TRMC instrument. The difference resonance frequency (Δf_0) is calculated from the f_0 when a TRMC cuvette is filled with neat toluene solvent and the f_0' when a TRMC cuvette is filled with the mixture of guest molecules and toluene, by $\Delta f_0 = f_0 - f_0'$. The shaded bands represent the 95% confidence range of the linear fitting results for visual guide, and linear fitting lines are depicted in gray color. (MeCN: acetonitrile (ε_r=36.6); *o*-DCB: 1,2-dichlorobenzene (ε_r=9.9); $CHCl_3$: chloroform (ε_r=4.81); CHX: cyclohexane (ε_r=2.02))

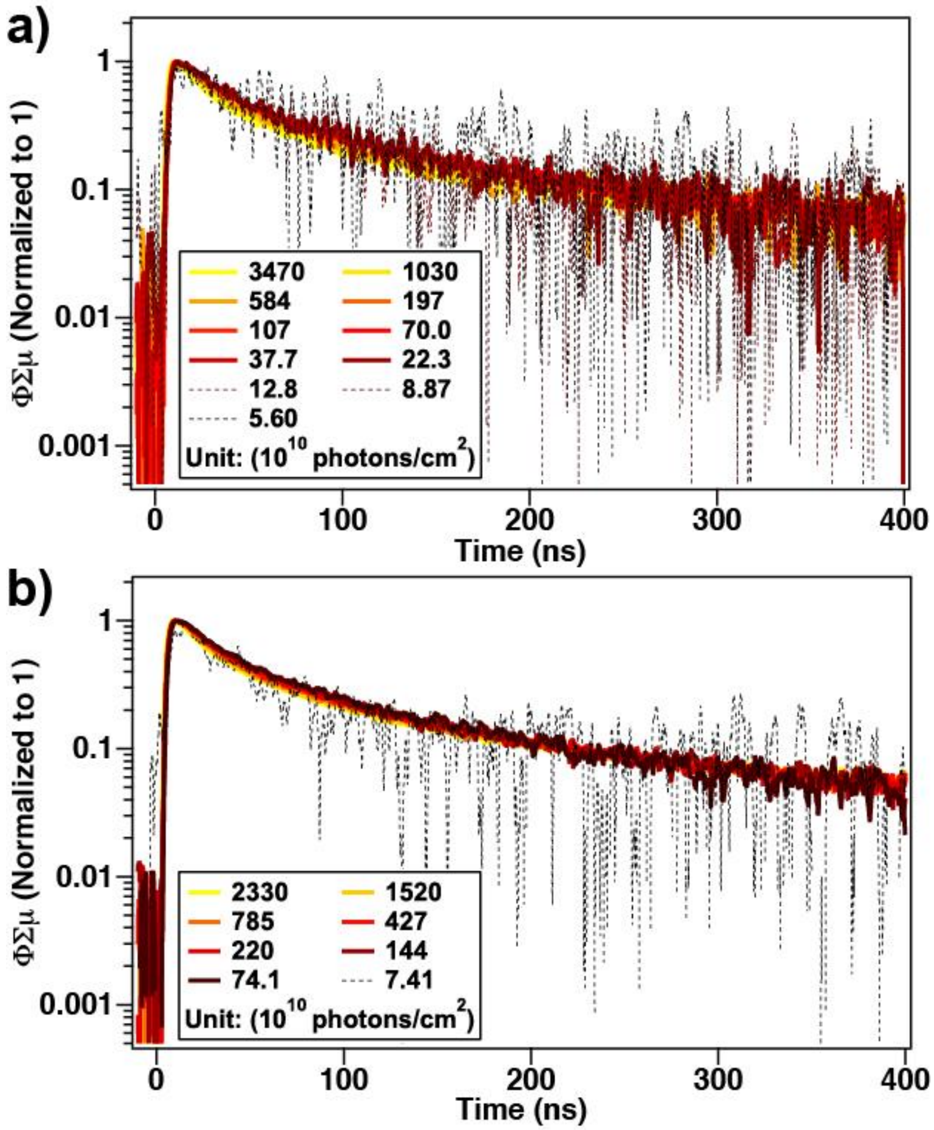

Supplementary Figure 9. The normalized yield-mobility product ($\Phi\sum\mu$) transient decay for (7,5)-SWCNTs upon a) S_{11} and b) S_{22} excitation measured at several excitation photon fluences noted.

Supplementary Methods

Materials Toluene (CHROMASOLV® grade, 99.9%) was purchased from Sigma-Aldrich and used as received. SWCNTs produced by the CoMoCat method were purchased from Southwest Nanotechnologies (SG65i, Lot no.:SG65i-L43), and poly[9,9-dioctylfluorenyl-2,7-diyl]-end capped with dimethylphenyl (PFO) was purchased from American Dye Source (ADS129BE, Molecular Weight = 40K—150K, Lot no.:14B016A1), and used as received. Acetonitrile (CHROMASOLV® grade, ≥99.9%), 1,2-dichlorobenzene (anhydrous, ≥99%), chloroform (anhydrous, ≥99%), and cyclohexane (CHROMASOLV® grade, ≥99.7%) were purchased from Sigma-Aldrich and used as received.

Instrumentation Electronic spectra of PFO and carbon nanotubes solution were recorded on a Varian 500 UV/Vis/NIR spectrophotometry system in either 2 mm or 10 mm quartz optical cells at ambient conditions.

Flash-Photolysis Time-resolved Microwave Conductivity Experiments (*fp*-TRMC) The details of *fp*-TRMC experimental setup and its theoretical background have been reported elsewhere.[1-4] However the present solution-phase *fp*-TRMC experiments require some explanation, as solution phase measurements have become quite uncommon. As stated in the text, *fp*-TRMC experiments measure the time-evolution of the complex dielectric constant of the sample after photoexcitation and the present experiments are conducted with the sample mounted in a microwave cavity, and the complex dielectric constant of the sample is calculated from the cavity resonance characteristics. Although the real part of the conductivity is identified with the imaginary part of the dielectric

constant – microwave absorption, it is important to realize that the imaginary dielectric constant can also have contributions unrelated to electrical conductivity: loss from dipole reorientation in the field. These two loss mechanisms (electrical conductivity and dielectric loss) are indistinguishable in our experiments. Indeed, most previous solution-phase *fp*-TRMC studies for small organic molecules dissolved in a low-dielectric solvent have focused on either dipole moment change or rotational relaxation of the solute upon photoexcitation, as the compounds studied do not generate mobile carriers.[5,6] In contrast, for carbon nanotubes suspended in solution microwave power loss is dominated by real conductivity on the tubes. The carbon nanotubes in our samples are very rigid structures and usually feature an average length in hundreds of nm to micron length scale. Thus the rotational frequency of carbon nanotubes in organic solvent is expected to be much lower than the GHz probe frequency and dielectric absorption in the nanotubes can be ruled out. In addition, given the energetics of the system (Figure 1b) and the oxidation potential of toluene ($E^{0/+}$ = 2.26 V vs SCE)[7] charge or energy transfer from the (7,5)-SWCNTs to the PFO or toluene solvent can be ruled out: the lowest energy state for excitons, electrons, and holes are all on the (7,5)-SWCNTs. Finally, we note that the high charge carrier mobilities typical of carbon nanotube species are expected to result in a large photoconductive response that would be likely to overpower any contributions from dielectric loss that may be present.

Calculation of Absorbed Photon Density in (7,5)-SWCNTs The absorbed photon density per micron length of (7,5)-SWCNTs is calculated via UV-vis-NIR absorption spectroscopy. Assuming that each absorbed photon will generate one exciton, the number of photons absorbed may be correlated with exciton density. For the absorption cross-

section at S_{11} transition for (7,5)-SWNTs, we used the molar extinction coefficient of 4,200 $M^{-1}cm^{-1}$ per carbon of (7,5)-SWCNTs from Weisman et. al.[8] As our excitation light is polarized, we calculated the effective concentration of illuminated (7,5)-SWCNTs in solution to account for excitation photoselection:[9] when a sample is excited with vertically polarized light, molecules with vertically aligned absorption transitions have the highest probability of excitation. The absorption probability is proportional to $\cos^2\theta$, where θ is the angle between the absorption dipole and z-axis. The average value of $\cos^2\theta$, $\langle\cos^2\theta\rangle$, is 3/5, and this 3/5 factor was applied to the original concentration to determine the effective concentration. Details of the exciton density calculations:

1) From the UV-vis-NIR absorption spectroscopy of the SWCNT solution, the SWCNT concentration was determined.

2) From the SWCNT concentration and the illuminated volume (V), the effective number of SWCNTs in the given V was obtained.

3) From the computed number of absorbed photons and the effective number of SWCNTs in V, the number of absorbed photons, namely excitons per micron length of SWCNT was calculated.

Finite Element Calculations of Microwave Photoconductance Sensitivity for a Solution Cell The use of microwave cavities to measure the dielectric and conductive properties of solid materials and solutions at GHz frequencies is well established.[10-15] However, quantitative interpretation of the data has always relied upon the use of sample and cavity geometries that are simple enough that analytical solutions to Maxwell's equations could be used to determine the sensitivity of the measurement. This has forced less than ideal experimental configurations, wherein an entire sample cavity is filled with

solution,[10,11] for example, necessitating contact between the solution and the metal walls that could lead to contamination.

In this work we use a more experimentally clean and convenient configuration, pictured in Supplementary Fig. 4a, consisting of a fused quartz cuvette that is mounted inside the waveguide cavity by embedding it in a Teflon block. In order to quantitatively evaluate the results we obtain in this relatively complex sample geometry, we resort to finite-element calculations of the electric field and power dissipation distributions to obtain a calibration factor that quantitatively relates our measured quantity (the microwave power reflection coefficient of the cavity at the resonant frequency) to the conductance of the sample.

Supplementary Figure 4b shows the electric field distribution obtained for this cavity configuration when pumped on resonance (~9.44 GHz), and Supplementary Fig. 4c shows distribution of power dissipation within the sample volume. At present, we use an average sensitivity over the entire sample volume, as in general the optical density of our samples is low enough for this approximation to be valid, but it is important to note the non-uniformity of the power dissipation, and account for it in cases where the optical density of the sample is high. In such a case the overlap between the distribution of excitations and power dissipation would need to be calculated, or simulations with a non-uniform change in sample conductivity implemented.

Supplementary Figure 5a shows the experimental cavity resonance curves along with the simulation results for these two cases. At lower frequency we see the curve for a cavity entirely filled with Teflon. At higher frequency we see the case of a cuvette mounted in the cavity filled with toluene. The simulated resonance curves were matched to the

experimental data by adjusting the dielectric properties of the simulated materials, where we find the dielectric properties of Teflon and toluene at ~9.5 GHz to be (ε_r = 2.045, δ = 7.5×10^{-5}) and (ε_r = 2.25, δ = 3.2×10^{-3}), which are in reasonable agreement with previous measurements at similar frequencies, where available.[14] The dielectric properties of quartz were taken to be: (ε_r = 3.75, δ = 1×10^{-4}). In each preceding case δ is the dielectric loss angle, where the complex permittivity is calculated as:

$$\varepsilon_r = \varepsilon_r' + i\varepsilon_r'' = \varepsilon_r'(1 - i\tan(\delta)) \qquad (1)$$

The sensitivity of the cavity to changes in sample conductivity is defined by:

$$\frac{\Delta P_r}{P_r} = -K\Delta G \qquad (2)$$

In order to obtain K from the simulation results, we simply simulate resonance curves for multiple different values of G and observe the change in reflected microwave power (ΔP) at the resonant frequency. Supplementary Figure 5b shows a plot of $\Delta P/P$ vs. ΔG over several orders of magnitude in sample conductance. We see that for small values of ΔG (< 10^{-4} S) the response is linear, and the slope provides the desired calibration factor: K = 2,400. For large values of ΔG this approximation of linear response is invalid, and the full simulated response curve must be used. All transient results reported in this paper adhere to the former criteria: ΔG < 10^{-4} S, and the value of K noted above is used throughout this work to calculate ΔG from measurements of $\Delta P/P$.

In order to verify that the finite-element simulations described above are accurate, and in agreement with previous methods for determining the sensitivity, we also present an analysis of a simpler cavity geometry: an idealized cell designed for measurement of solid films. Supplementary Figure 6a shows the electric field distribution for this configuration, consisting of a simple rectangular waveguide cavity with a thin-film

sample mounted at one of the two electric field maxima. Supplementary Figure 6b shows the resulting simulated resonance curves for ΔG = 0 and 10^{-6} S. An analysis identical with the one performed for the solution cell (Supplementary Fig. 6c) shows that K = 32,000 in this configuration. Previous work has used an analytical relationship between the Q-factor of a loaded microwave cavity and the sensitivity in this geometry. This relationship is:[16,17]

$$K = \frac{2Q_l(1+1/\sqrt{R_0})}{\pi f_0 \varepsilon d a/b} \qquad (3)$$

where Q_l is the loaded quality-factor of the cavity, R_0 is the microwave power reflection coefficient on resonance, f_0 is the resonant frequency, d is the thickness of the sample along the microwave propagation axis, and a and b are the long and short dimensions of the waveguide cross section, respectively.

Fitting the simulated resonance curve with a Lorentzian function yields a Q_l =200, and substituting this into Supplementary Eq. (3), along with f_0 and R_0, provides K = 32,900, an error of 3% relative to the true value from the simulation.

It is worth pointing out the marked difference in sensitivity between the solid-film configuration, and the solution cell configuration. The K-factor is much lower for the latter. There are several reasons for this. First, because of dielectric losses in the Teflon, quartz, and toluene present in the solution configuration, the Q-factor of that cavity is much smaller than that for thin-film samples: 80 vs. 200. Alone, this difference accounts for a factor of ~2.5 reduction in sensitivity. Second, because we define sensitivity in terms of conductance, the large extent of the sample in the solution configuration serves to reduce sensitivity further; in the thin-film configuration the entire sample is concentrated at the electric field maximum, and extends over the entire cross section of

the waveguide. In contrast, the longitudinal dimensions of the solution cell are ~¼l, and thus much of the sample is subject to much lower electric fields. This is most evident in the distribution of power dissipation (proportional to the square of the electric field) in Supplementary Fig. 4c. Moreover, the solution cell only fills half the total short-wall dimension of the waveguide. Combined, these two effects can be expected to reduce sensitivity by an additional factor of ~4. This simple analysis shows that the order of magnitude difference in K observed between the thin-film and solution-cell configurations is intuitively reasonable.

All simulations were performed in COMSOL Multiphysics 4.4 using the RF module. All aspects of the cavity interior were modeled explicitly. The walls of the waveguide were represented by an impedance boundary condition, with a conductivity of 1.7×10^{7} S m^{-1} (suitable for brass). The input microwave power was set to 11 mW, and coupled into the model using a "Port" boundary condition at the open end of the feed waveguide. The microwave power reflection coefficient of the cavity (P_{R11}) was determined using the scattering parameter, Γ_{11}, calculated for the input port and calculated as:

$$P_{R11} = |\Gamma_{11}\Gamma_{11}^{*}| \qquad (4)$$

where Γ_{11}^{*} is the complex conjugate of the scattering parameter.

The optical coupling holes were modeled by positioning a large block of "lossy air" outside the cavity, so that any microwave power radiated through the holes (and a very small amount *is* radiated) will be absorbed before reaching the edge of the simulation domain, and not be reflected back, effectively simulating radiation into an infinite external volume.

Microwave Cavity Resonance Measurements as a function of a guest molecule:toluene mass ratio As shown in Supplementary Eq. (1), when an electromagnetic wave propagates in a dielectric medium, the complex permittivity determines the phase-shift of the wave with respect to that in vacuum by the dielectric constant (ε'_r) and the attenuation of the wave amplitude by the dielectric loss (ε''_r). Therefore, using the properties of the microwave phase-shift as a function of the dielectric constant, we can make a relative comparison of the dielectric constant of PFO with other compounds to assess the local dielectric environment of PFO-wrapped nanotubes. In our case, using a resonant cavity to contain the sample, we can very sensitively gauge the real part of the dielectric constant from the resonance frequency of the loaded cavity. Supplementary Figure 8 displays the difference resonance frequency (Δf_0) that is calculated from the f'_0 when a TRMC cuvette is filled with neat toluene solvent and the f''_0 when a TRMC cuvette is filled with the mixture of guest molecules (MeCN: acetonitrile (ε'_r=36.6); *o*-DCB: 1,2-dichlorobenzene (ε'_r=9.9); $CHCl_3$: chloroform (ε'_r=4.81); CHX: cyclohexane (ε'_r=2.02); PFO) and toluene, by $\Delta f_0 = f'_0 - f''_0$. To produce a resonance frequency shift of −0.05 GHz, MeCN:toluene needs only ~0.8% (weight), *o*-DCB:toluene needs ~6.5% (weight), and chloroform needs ~21% (weight). In contrast, the resonance frequency (f''_0) in a PFO:toluene mixture does not change with increase of PFO mass ratio, suggesting the dielectric constant of PFO is comparable to that of toluene, and is clearly less than that of chloroform.

Supplementary References

1. de Haas, M. P. & Warman, J. M. Photon-induced molecular charge separation studied by nanosecond time-resolved microwave conductivity. *Chem. Phys.* **73,** 35–53 (1982).
2. Schins, J. M. & Talgorn, E. Conductive response of a photo-excited sample in a radio-frequent driven resonance cavity. *Rev. Sci. Instrum.* **82,** 064703 (2011).
3. Saeki, A., Koizumi, Y., Aida, T. & Seki, S. Comprehensive approach to intrinsic charge carrier mobility in conjugated organic molecules, macromolecules, and supramolecular architectures. *Acc. Chem. Res.* **45,** 1193–1202 (2012).
4. Savenije, T. J., Ferguson, A. J., Kopidakis, N. & Rumbles, G. Revealing the Dynamics of Charge Carriers in Polymer:Fullerene Blends Using Photoinduced Time-Resolved Microwave Conductivity. *J. Phys. Chem. C* **117,** 24085–24103 (2013).
5. Warman, J. M., de Haas, M. P., Grätzel, M. & Infelta, P. P. Microwave Probing of Electronic Processes in Small Particle Suspensions. *Nature* **310,** 306–308 (1984).
6. Piet, J. J., Taylor, P. N., Anderson, H. L., Osuka, A. & Warman, J. M. Excitonic Interactions in the Singlet and Triplet Excited States of Covalently Linked Zinc Porphyrin Dimers. *J. Am. Chem. Soc.* **122,** 1749–1757 (2000).
7. Merkel, P. B., Luo, P., Dinnocenzo, J. P. & Farid, S. Accurate Oxidation Potentials of Benzene and Biphenyl Derivatives via Electron-Transfer Equilibria and Transient Kinetics. *J. Org. Chem.* **74,** 5163–5173 (2009).
8. Streit, J. K., Bachilo, S. M., Ghosh, S., Lin, C.-W. & Weisman, R. B. Directly Measured Optical Absorption Cross Sections for Structure-Selected Single-Walled Carbon Nanotubes. *Nano Lett.* **14,** 1530–1536 (2014).
9. Lakowicz, J. R. *Principles of Fluorescence Spectroscopy*. (Springer, 2007).
10. Warman, J. M., de Haas, M. P. & Hummel, A. Detection of Electrons in Pulse Irradiated Liquid Hydrocarbons by Microwave Absorption. *Chem. Phys. Lett.* **22,** 480–483 (1973).
11. de Haas, M. P. & Warman, J. M. Photon-Induced Molecular Charge Sparation Studied by Nanosecond Time-Resolved Microwave Conductivity. *Chem. Phys.* **73,** 35–53 (1982).
12. Warman, J. M., de Haas, M. P. & Wentinck, H. M. The study of radiation induced conductivity changes in microheterogeneous materials using microwaves. *Int. J. Radiat. Appl. Instrum. C Radiat. Phys. Chem.* **34,** 581–586 (1989).
13. Fessenden, R. W., Carton, P. M., Shimamori, H. & Scaiano, J. C. Measurement of the dipole moments of excited states and photochemical transients by microwave dielectric absorption. *J. Phys. Chem.* **86,** 3803–3811 (1982).
14. Krupka, J., Derzakowski, K., Riddle, B. & Baker-Jarvis, J. A dielectric resonator for measurements of complex permittivity of low loss dielectric materials as a function of temperature. *Meas. Sci. Technol.* **9,** 1751–1756 (1998).
15. Sheen, J. Study of microwave dielectric properties measurements by various resonance techniques. *Measurement* **37,** 123–130 (2005).
16. Infelta, P. P., de Haas, M. P. & Warman, J. M. Study of Transient Conductivity of Pulse Irradiated Dielectric Liquids on a Nanosecond Timescale Using Microwaves. *Radiat. Phys. Chem.* **10,** 353–365 (1977).
17. Dicker, G. Photogeneration and dynamics of charge carriers in the conjugated polymer poly (3-hexylthiophene). Ph. D. Dissertation. (Delft University of

Technology, 2004).
